# Fast Computation and Applications of Genome Mappability

**DOI:** 10.1371/journal.pone.0030377

**Published:** 2012-01-19

**Authors:** Thomas Derrien, Jordi Estellé, Santiago Marco Sola, David G. Knowles, Emanuele Raineri, Roderic Guigó, Paolo Ribeca

**Affiliations:** 1 Institut de Génétique et Développement (IGDR), Université Rennes 1, Rennes, France; 2 Centro Nacional de Análisis Genómico (CNAG), Barcelona, Spain; 3 Centre for Genomic Regulation (CRG), Universitat Pompeu Fabra, Barcelona, Spain; The Centre for Research and Technology, Hellas, Greece

## Abstract

We present a fast mapping-based algorithm to compute the mappability of each region of a reference genome up to a specified number of mismatches. Knowing the mappability of a genome is crucial for the interpretation of massively parallel sequencing experiments. We investigate the properties of the mappability of eukaryotic DNA/RNA both as a whole and at the level of the gene family, providing for various organisms tracks which allow the mappability information to be visually explored. In addition, we show that mappability varies greatly between species and gene classes. Finally, we suggest several practical applications where mappability can be used to refine the analysis of high-throughput sequencing data (SNP calling, gene expression quantification and paired-end experiments). This work highlights mappability as an important concept which deserves to be taken into full account, in particular when massively parallel sequencing technologies are employed. The GEM mappability program belongs to the GEM (GEnome Multitool) suite of programs, which can be freely downloaded for any use from its website (http://gemlibrary.sourceforge.net).

## Introduction

Dramatic advances in massively parallel sequencing technologies (also known as *high-throughput sequencing*, HTS or *next-generation sequencing*, NGS) have extended the application of sequencing of nucleic acid to many problems in genomics, epigenomics, and transcriptomics. Whole or targeted genome sequencing and re-sequencing, genome-wide profiling of chromatin status and transcription factor binding through ChIPSeq, quantitative estimation of transcriptome abundance through RNASeq, metagenomic and metatranscriptomic sequencing are a just a few examples. HTS technologies typically produce huge amounts of short sequences (*sequence reads*, or simply *reads*), with different lengths and characteristics (qualities, error rates, structural designs, etc.) depending on the technology employed. A fundamental step of the post-sequencing protocol often consists in efficiently aligning the sequence reads onto a genome used as reference. Numerous short read aligners (or *mappers*) have been developed to achieve this goal with suitable efficiency [1], [2]. However, owing to the non-random nature of genomes –which include a significant proportion of structurally, evolutionary and functionally important repetitive sequences [3], [4]– one key part of the mapping step is to discern reads that can be aligned to only one single location (*uniquely mapping* reads) from reads matching multiple possible locations in the reference (*multiply mapping* reads). The latter are often interpreted differently by existing mappers, and sometimes even altogether discarded during the analysis steps subsequent to mapping. The specific interpretation of multiply mapping reads may have implications on the outcome of downstream analysis, in particular when reads are used to obtain quantitative estimates. Typical examples are the determination of transcription factor binding affinity (in ChIPSeq experiments) or transcript abundance (in RNASeq experiments). Thus, for a given HTS run one would aim at maximizing the number of unique mappings obtained, in order to exploit most of the signal from the biological input.

For a given genome, the proportion of uniquely mapped reads depends mostly on the length of the sequence reads produced by the experiment, and on the number of mismatches allowed during the mapping step [5]. Therefore, given the technical specifications of the sequencing experiment it is possible to compute *a priori* the mappability of the whole sequence, i.e., the inverse of the number of times that a read originating from any position in the reference genome maps to the genome itself – thus identifying, for instance, the regions that are truly “mappable”, that is those producing reads which map back unambiguously to themselves. Regions with high mappability will tend to produce unique mappings, while regions with low mappability will tend to produce ambiguous mappings. Mappability information can therefore be used *a priori*, as a guide to fine-tune the design of an HTS experiment in order to increase the number of uniquely mappable reads.

In addition, the mappability information is crucial when quantitative estimates are produced. Indeed, mappability has been originally introduced and used to carry out quantitative studies of binding affinity from ChIPSeq experiments, where the ratio of input versus control peaks cannot be exactly assessed unless the proper normalization factor is known [6]. In these studies the mappability of various genomes has been investigated without considering mismatches. However, one must make provision for sequencing errors inherent to HTS technologies, as well as for polymorphisms or variants between the individual genome/transcriptome actually sequenced and the genome used as a mapping reference. Therefore, it is customary to allow for mismatches when mapping reads; the 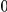-mismatch mappability is not sufficient in these cases. Unfortunately, computing the mappability of a sequence up to even a few mismatches is usually a task orders of magnitude more expensive than when no mismatches are allowed, and hence not one routinely performed.

In this work, we introduce and describe a mapping-based method to compute the mappability of an entire sequence of the size of a mammalian genome up to an arbitrary number of mismatches, which is guaranteed to be comparatively fast even for short reads and very redundant sequences. The method produces the exact mappability in the case of 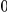 mismatches, and a very good approximation of it if a non-zero number of mismatches is allowed. For different number of substitutions, we then examine the genome-wide mappability profiles of four model organisms (human, mouse, fly and nematode), for which we also produced visualization schemes as part of the UCSC genome browser [7].

Second, we study the mappability of the transcribed genomic regions. Since a high proportion of transcripts in a genome exhibit repetitive sequences –such as repeated functional units or retrotransposons (LINEs and SINEs)– which can influence their mappability profiles, we use our mappability method to explore the variations in mappability of different classes of transcripts, taking the GENCODE annotation [8] as a reference. We show that indeed mappability profiles vary greatly with the transcript class (protein-coding genes, non coding RNAs, orthologous families, etc). We thus propose an improved measure of RNA quantification which takes into account the mappability at the level of the single locus.

Third, we investigate how the use of paired-end sequencing or mate-pair libraries relates to mappability. To this end, we predict and quantify how many of the pairs obtained from a typical DNASeq experiment can be rescued by taking advantage of the uniqueness of one of its reads and of the distance information for the pair.

In conclusion, we are able to precisely link our findings to the design of a better experiment when the focus is on some particular element in the genome. Our results suggest that the mappability is an important concept to be taken into account when one is trying, for instance, to re-sequence a particular genomic region, or to produce quantitative estimates of transcript abundance from RNASeq experiments.

## Methods

Formally, our definition of the mappability is the following. Given some read length 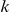, the 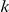 *-frequency* 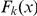 of a sequence at a given position 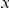 corresponds to the number of times the 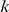-mer starting at position 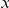 appears in the sequence and in its reverse complement, considering as equivalent all the 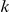-mers which differ by less than some predefined alignment score (like a given number of mismatches–for the sake of simplicity, in the rest of this paper we will assume a framework where only substitutions, and neither insertions nor deletions, are allowed during alignment). For instance, the 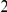-frequency up to 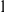 substitution of the string TICTACTOE at positions 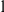 to 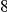 is given by the values 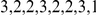. It is possible to define an analogous quantity, the 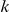 *-mappability* or 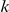 *-uniqueness* 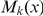, as the inverse of the frequency: 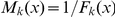. While the frequency usually varies by several orders of magnitude, the mappability has the advantage of always being a quantity between 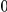 and 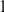, and such that the highest possible values of 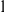 correspond to uniquely mapping position (here, and throughout all the paper, by “unique” we mean “unique up to the specified number of mismatches”).

Various methods can be employed to compute the exact frequency (and hence the mappability) of a sequence. The simplest one is a brute-force approach, consisting in the explicit enumeration and counting of all the 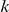-mers present in the sequence; it is practical only for very short strings. More sophisticated strategies might rely on the traversal of some string data structure –like a suffix tree, a suffix array or a hash table– to directly obtain an enumeration of the 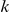-mers together with their counts; this is what has been used in [5], [6]. All such approaches, however, become problematic if mismatches are allowed: the authors of [6] report for their method a slowdown of a factor of 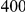 when 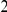 substitutions are permitted, and they do not go further than evaluating the mappability of just 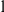 Mb of the human genome using such an edit distance.

In the case of mismatches, essentially, the problem of computing the frequencies becomes equivalent to that of exhaustively mapping all the positions in the sequence after a suitable choice of the alignment parameters. At a first glance, this goal would seem well within the range of existing high-performance mappers, which may easily attain speeds of several tens of millions of mapped reads per hour; assuming a mapping speed of 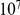 reads per hour, for instance, it would appear possible to compute the frequencies of the human genome in only about 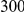 hours, with the additional possibility of distributing the computation among different processors.

There are two algorithmic issues, however. The first one is that most of the available mappers are not based on exhaustive alignment algorithms; this fact implies that they are unable to report the exact count of all the existing matches for a given sequence (although they can usually return a more or less precise approximation of such a quantity). The second problem concerns performance: most mapping algorithms are usually optimized to quickly report a few matches and most of them become (much) slower when requested to perform full counting queries. In practice, the speed of all implementations of mapping algorithms always shows some dependency on the number of matches found in the reference; this means that aligning one million reads which all map to thousands of locations in the genome will be (much) slower than aligning one million reads mapping uniquely. Performance degradation may become very relevant in the case of a brute-force enumeration of a sequence in a genome (for instance, it is still possible to find in *H.sapiens* genomic locations having 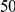-mappability as large as 10,000 when 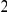 substitutions are allowed – like ACGGTGGCTCATGCCTGTAATCCCAGCACTTTGGGAGGCCGAGGCGGGCG, which appears 15,323 times with less than 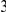 nucleotide substitutions). In general, performance will be worse when the mappability of a transcriptome is being evaluated, and dramatically worse when small values for 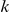 are used, as in typical ChIPSeq or MNaseSeq experiments.

In our framework, the first problem is automatically taken care of by our own genome indexing implementation, which provides for fast exhaustive searches and counting queries [2]. We address the second issue by noting that most of the degradation in performance actually comes from the fraction of 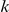-mers showing high frequencies, where thousands of 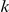-mers exist which are equivalent within the specified number of mismatches; thus, most of the computational time is actually spent mapping such set of 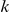-mers over and over again, each time any single element of the set is mapped.

To avoid the latter problem at least in part, we perform the following approximation: each time a 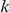-mer is mapped within the given number of mismatches to a set of positions 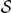, one can pretend that all the positions in 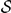 have already been mapped, assign to them a frequency value equal to the number of elements in 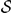, and skip them altogether from that point on. Such a strategy is not enough to completely factor the redundancy out –it is effective in eliminating only the equivalent 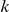-mers occurring in the sequence *after* the 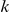-mer being mapped–, and is only exact for 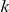-frequencies when no substitutions are allowed. From a practical standpoint, however, the mappability computed in this way is a good approximation of the exact mappability, and, more importantly, is computationally feasible even when 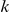 is small. The complete algorithm is as follows.

**Algorithm 1 (Fast mappability computation)** *To compute the* 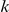 *-frequencies of a sequence of length* 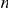 *up to* 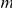 *mismatches, given an approximation parameter* 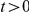 *:*

1. ***initialize and zero*** *an array* 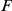 *of* 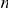 *numbers*
2. ***for*** *all positions* 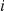 *in the sequence* ***do***

***if*** 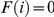 ***then***

*(a) take the* 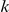 *-mer* 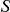 *starting at position* 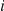


*(b) compute all the positions* 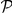 *in the sequence to which* 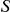 *maps up to* 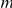 *mismatches*

*(c)* ***set*** 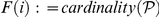


*(d)* ***if*** 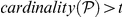 ***then***

***for*** *all positions* 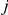 *in* 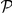 ***do***

***if*** 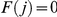 ***then***

***set*** 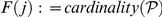


***else***

***set*** 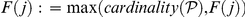

1. ***output*** *the array* 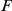.

When 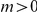, the proposed algorithm provides approximated values for the frequency of positions which are not unique in the sequence: this is due to the fact that, given two different 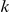-mers 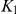 and 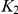, the set 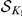 of locations equivalent to 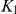 up to the given number of mismatches is in general different from the set of locations 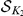 equivalent to 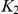; hence, considering 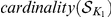 as the frequency of both 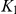 and 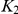 is an approximation. However, as stated above the algorithm is acceptable from a practical standpoint since:

1. it is exact for the whole sequence when 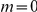

2. when 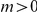 it still gives correct values for the frequency of the 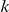-mers that are unique within the specified number of mismatches, as a 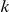-mer 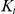 can belong to the set 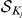 of locations equivalent to a previously occurring 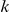-mer 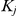 only if it is not unique; in addition, the parameter 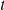 allows to propagate an approximated frequency value only when it is sufficiently large
3. the difference between approximated and real frequency can be large in absolute terms, but in the majority of cases it represents only a relatively small fraction of the correct value, since the optimization affects the locations in proportion to their redundancy.

Another important observation is about the presence of the maximum function in the *else* branch of case (2d): this choice regulates the case when a 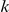-mer is hit more than once during the mapping of other similar 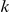-mers. Since the frequency of the 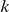-mer will not be directly recomputed due to the chosen optimization strategy, the maximum of all the possibilities is taken as its value, to make sure that an underestimation of the actual value is avoided as much as possible.

Finally, we emphasize that when the exact mappability is needed the approximation can always be turned off by setting 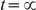: this produces exact frequencies, albeit at the price of possibly much longer running times if 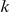 is small and/or the genome is very repetitive.

We made use of the latter property to test our approximation, and assess how well it correlates with the exact results. To this end we performed two runs for each example, one with the value of 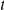 automatically selected by the program, and another with 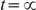; we then compared the results thus obtained.

**Figure 1:**
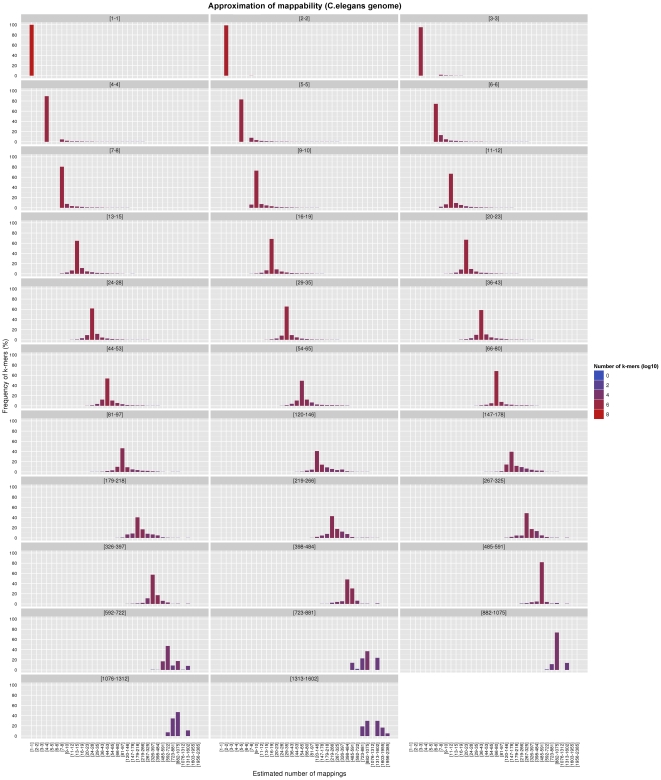
Effect of our approximation on the frequencies of the *C.elegans* genome, for 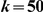
 **and** 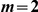. Both the exact and the approximated data were obtained with gem-mappability, the former by setting the value of parameter 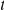 to 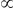, the latter with the default value of 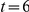 automatically selected by the program after the length of the *C.elegans* genome. Each panel shows how our approximation scatters the 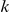-mers originally populating a non-approximate 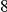-bit frequence bin into more than one single approximate bin. Using the panel [9]–[10] as an example, one can see that about 80% of the 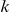-mers fall into the correct bin, while the remaining 20% is dispersed in bins from [7]–[8] to [24]–[28], with most of the 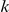-mers staying in bins close to the correct one. In addition, the color of the bins shows that such a 20% of 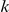-mers corresponds in absolute terms to a small number (in this example about the 90% of the 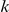-mers of the genome is unique and hence falls into the [1–1] bin, which, as explained in the text, is not perturbed by our approximation owing to the good properties of the latter).

**Figure 2:**
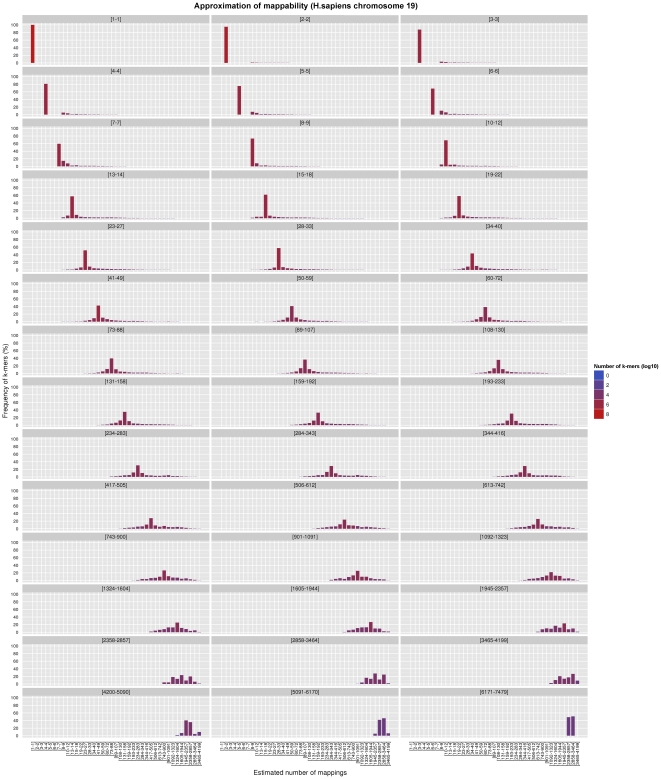
Effect of our approximation on the frequencies of chromosome 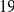
 **of** ***H.sapiens*** **, for** 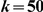 **and** 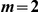. Both the exact and the approximated data were obtained with gem-mappability, the former by setting the value of parameter 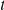 to 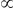, the latter with the default value of 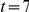 automatically selected by the program after the length of chromosome 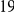 of *H.sapiens*. Each panel shows how our approximation scatters the 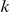-mers originally populating a non-approximate 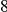-bit frequence bin into more than one single approximate bin.

We applied such a procedure to both the complete genome of *C.elegans* (using the default value of 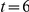 versus 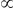, see Figure 1) and the chromosome 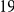 of *H.sapiens* (with the default 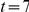 versus 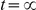, see Figure 2); being the richest of its genome in segmental duplications and paralog genes (the 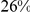 of the sequence, with respect to a whole-genome 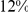 average), human chromosome 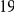 is particularly suited as a test on the scale of a mammalian chromosome. In both cases we chose an intermediate 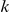-mer size of 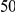 bp. Each panel of the aforementioned Figures focuses on a different set of 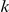-mers, all those belonging to the same frequency bin in terms of our 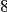-bit reduced-precision representation of the mappability (see next Section) when no approximation is applied. In case of exact computation, such 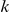-mers will all fall into one single bin in their respective panel; when the approximation is active, however, some 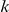-mers will migrate to other bins of the same panel, since their estimated frequency is now different from the actual value – and the better the approximation, the more the 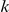-mers staying in their correct bin.

As expected, both Figures show that the approximation is good: in each panel the distribution of 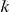-mers is usually centered around the correct frequency value, and the number of approximated values which are very overestimated or very underestimated is uniformly low. Furthermore, 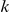-mers having frequencies of less than 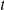 only appear in their respective bins, as they should after our thresholding rule. However, one can better appreciate the value of the approximation only taking into account another important point: in general, the number of genome positions having a frequency above the threshold 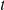 is very low (in the case of *C.elegans*, less than 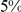; less than 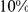 for human chromosome 19); hence, the number of incorrectly estimated high-frequency genomic locations will be even lower, and small in absolute terms. In the case of human chromosome 19 and 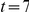 illustrated in Figure 2, for instance, almost the 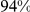 of the 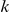-mers maintain their correct frequency bin after approximation; and more than the 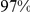 fall within 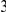 bins of distance from the correct one. It should also be mentioned that, being mappability defined as the inverse of the frequency, an underestimation/overestimation of the frequency at a very redundant genomic location will not result in large differences in the value of the mappability for that location, since both the true and the estimated value will be large. We concluded that our approximation is sound for most practical uses.

Finally, it is evident that the proposed algorithm is still easily distributable, in particular in a multi-threaded shared-memory model (so far, a multi-core parallelization has proven sufficient for all of our computations).

### Implementation

The algorithm presented in the previous Section has been implemented on top of the GEM (GEnome Multitool) library for the indexing of HTS data [2]. The library provides a very fast C mapping engine, based on the Burrows-Wheeler Transform [9] and custom mapping algorithms (whose description is out of the scope of this paper, and will be presented elsewhere). The C library can be accessed via various interfaces written in higher-level programming languages, notably Objective Caml [10]; such interfaces allow to prototype and implement new algorithms in a concise way.

One relevant difference between the algorithm of last Section and our implementation is that we chose to encode the frequency array as reduced-precision 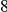-bit numbers, each one representing a range of frequency values; low numbers encode a single value (frequency equal to 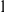, 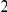, and so on) while higher numbers represent larger and larger intervals. Although different choices might have been possible, this solution has the clear advantage of providing a consistent reduction in memory consumption. In addition, storing the frequency values at full resolution is hardly useful, since we are usually interested in knowing the exact frequency only when it is small (typically values ranging from 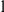 to 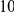), while we can usually get by with its approximate value when it is in the range of the hundreds, thousands or more. The results are output as a pseudo-multi-FASTA file, where the frequency at each position is encoded as a printable ASCII character.

Finally, we note that along with the mappability program we also provide a fast retriever; such a program allows the user to pre-index the obtained mappability files (which are huge), and to subsequently query them in an efficient way.

#### Benchmarks

We evaluated the program on an Intel machine with 8 Xeon X5570 CPU cores clocked at 3 GHz, using 8 cores for all the tests. Such a number of processors has been chosen to reduce the wall-clock running time as much as possible; however, it is likely to be suboptimal, since due to the competition in memory access the efficiency of shared-memory distributed computation decreases when many threads are used.

Typical running times and memory occupancy for various genomes are shown in Table 1. When the approximation is active, the whole-reference mappabillity can usually be computed within a few hours. On the other hand, in the bottom-most panel one can find the timings obtained when setting the parameter 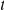 to 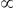: this has the effect of turning off our approximation, and is indeed equivalent to performing a brute-force mapping of all the 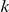-mers in the genome. For the *H.sapiens* genome, such a method is several times slower than the approximate one at 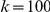, and completely unfeasible at 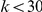, thus confirming that the approximation is necessary when 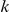 is small. On the other hand, a brute-force approach might be practically feasible at large 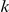, albeit at the price of much longer running times.

**Table 1:** Performance of gem-mappability for different parameters and genome species.

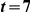			*D.melanogast.*		*D.melanogast.*	
			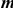 = 2, 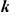 = 36		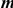 = 3, 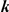 = 75	
Time			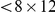 mins		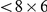 mins	
Mem.			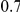 GB		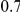 GB	
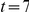	*H.sapiens*	*H.sapiens*	*H.sapiens*	*H.sapiens*	*H.sapiens*	*H.sapiens*
	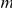 = 0, 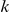 = 36	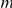 = 1, 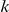 = 24	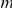 = 2, 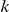 = 36	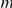 = 2, 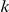 = 50	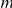 = 3, 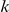 = 75	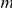 = 4, 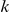 = 100
Time	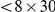 mins	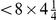 hrs	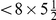 hrs	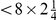 hrs	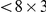 hrs	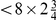 hrs
Mem.	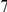 GB	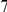 GB	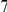 GB	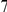 GB	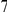 GB	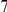 GB
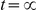			*H.sapiens*	*H.sapiens*		*H.sapiens*
			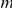 = 2, 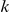 = 36	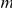 = 2, 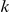 = 50		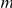 = 4, 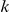 = 100
Time			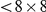 days	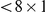 day		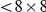 hrs
Mem.			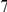 GB	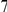 GB		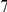 GB

We used 8 cores for all the tests. Wall-clock times are reported. In the lowest panel, the timings are those obtained when computing the exact mappability (i.e., with our approximation switched off).

#### Visualization of mappability

The mappability scores produced by our program can be easily converted to other formats, like those suitable for display in the UCSC genome browser [7]. To this end, we just have to compute the mappability at each position out of the corresponding frequency. We note again that the mappability naturally lends itself to a good visualization, since it always has a value between 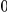 and 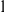, with the highest scores corresponding to uniquely mapping positions. On the other hand, the direct visualization of frequency would be problematic: its dynamic range is large –with values possibly varying from 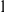 to millions–, but at the same time the most interesting region is usually that of small values.

**Figure 3:**
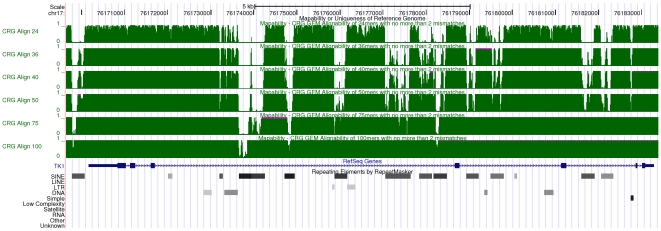
Visualization of mappability on the UCSC browser [**7**]: the example of the human TK1 gene. Six mappability tracks (green) are shown here corresponding to 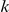-mer sizes 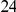, 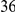, 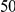, 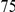 and 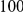 bp (from top to bottom of the figure). Regions with low mappability score have high frequencies, and conversely. This example illustrates that the uniqueness of the TK1 locus (especially within the introns) could be inversely correlated with the presence of some repetitive elements as identified by RepeatMasker [37].

We have created mappability tracks for multiple genome species and annotations, including human (both assembly versions Hg18 and Hg19/GRCH37) and mouse (mm9) genomes. At the time of this writing such tracks are integrated in the official UCSC genome browser (see Figure 3), and hence can be immediately explored on-line without the need of any further preparation from the user. For other organisms –mainly *D.melanogaster* (dm3) and *C.elegans* (ce6)– we also generated additional tracks which are not part of the UCSC genome browser; they can be obtained from the authors upon request, and subsequently visualized as custom tracks. In case no precomputed track is available for some organism, the users can easily compute it on their own even on modest hardware.

## Results

### The mappability of eukaryotic genomes

Given a random sequence of length 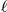 drawn from an alphabet of 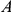 symbols, we define its *proper length* 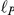 as the shortest length such that any string of length 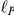 is expected to occur in the sequence about once, that is 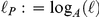. For instance, the proper length for the human genome is 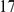 (since 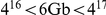), while that of *D.melanogaster* is 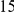, and 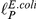 only 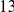. Hence, for any sequencing read length 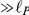 (a condition already satisfied even by “short” sequence reads of 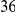 bp produced by early versions of some HTS technologies) one would expect the genome mappability to be 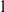 almost everywhere – that is, that most reads map uniquely.

However, this is often not the case. There are two main reasons for this deviation from the expected behavior:

1. genomes are far from being random; they are the result of a long evolutionary history that includes frequent duplications, involving the whole genome or specific regions [11]. The result is a fractal-like structure with repetitions of different nature [12] appearing at different levels of resolution [13] – from large structural variants, including segmental duplications [14], to copy number variations [15], long and short interspersed repeats, paralogous gene families, pseudogenes, and modular domains appearing within the sequence of functionally diverse genes [16]
2. mismatches are often allowed when mapping HTS reads, and hence in mappability computations. As a result, they lower the number of unique 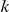-mers in the genome.

Even relatively long sequences may map to multiple locations, in particular when mismatches are allowed. Using our method, we have sought to characterize the mappability profiles at a whole genome level for four model organisms (human, mouse, fly, nematode): in Table 2 we extend the results obtained in [6] –where the mappability was computed with 0 mismatches– by listing the number of uniquely mapping positions in the case of 2 substitutions for three arbitrarily defined read lengths (36, 50 and 75 bp) frequently used in HTS experiments. As expected, mappability correlates with sequence length and number of mismatches: the longer the reads and the smaller the number of mismatches, the higher the uniqueness of the sequence reads. It is interesting to note that with the parameters typically used in ChIPSeq experiments (36-bp reads and two substitutions) a large fraction of eukaryotic genomes is not uniquely mappable: in principle, even exact sequence reads obtained from such loci cannot be inequivocally assigned to their originating positions. As we have already pointed out, this has obvious important implications for quantitative estimates (for instance, transcription factor binding affinity or intensity of chromatin modifications). This fraction represents 30% of mammalian and insect genomes at 36 bp; extending the read length increases the uniquely mappable fraction, but even with longer reads of 75 bp (and maintaining constant the number of substitutions, that is effectively increasing the stringency of the mapping) almost 20% of the human genome remains unmappable. Even a very restrictive prescription which requires exact mapping (0 mismatches) of 75-bp sequence reads leaves 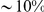 of the human genome unmappable.

**Table 2:** Relationship between the proportions/type of repeat elements and the proportion of 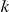-mers having a mappability score of 1 (i.e., uniquely mappable).

		*H.sapiens*	*M.musculus*	*D.melanogaster* (dm3)		*C.elegans*
		(hg19)	(mm9)	with het.	without het.	(ce6)
Genome size (bp)		3,107,677,273	2,725,765,481	168,736,537	159,454,756	100,281,426
Repeat sequences (bp)		1,406,290,513	1,153,714,659	44,719,009	38,601,028	13,121,257
Proportion of repeats		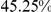	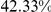	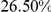	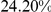	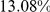
LTR		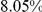	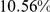	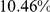	–	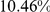
Non-LTR	SINEs	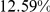	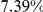	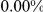	–	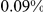
	LINEs	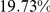	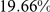	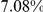	–	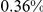
Uniquely mapped positions ( 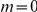 )						
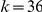		2,489,885,654	2,178,433,024	119,915,412	116,918,511	92,332,303
		( 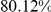 )	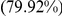	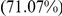	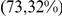	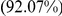
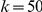		2,627,947,484	2,267,226,534	121,732,432	118,368,697	93,775,749
		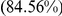	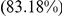	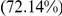	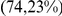	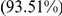
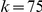		2,729,902,459	2,349,591,487	124,087,375	120,329,119	95,226,461
		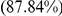	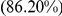	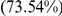	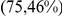	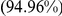
Uniquely mapped positions ( 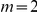 )						
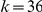		2,175,066,863	1,964,593,763	114,889,241	113,088,604	87,385,879
		( 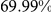 )	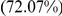	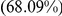	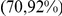	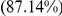
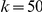		2,380,109,920	2,100,436,231	117,178,560	114,915,550	90,050,144
		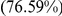	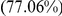	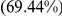	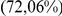	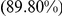
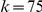		2,582,297,225	2,225,670,208	119,798,046	116,955,098	92,369,340
		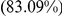	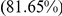	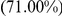	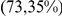	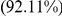

Repeat elements have been identified and classified by the RepeatMasker program [37]. The mappability has been computed for 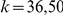 and 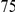, with 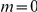 and 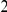.

While there is a negative correlation between the uniqueness of the genome and its repetitive content, the relationship between genome structure and mappability is more complex. For instance, while the proportion of repeats in the *D.melanogaster* genome is lower than that of mammalian genomes, the fraction of its mappable genome is not larger. Interestingly, in the fly genome, in contrast with the other genomes analyzed, uniqueness does not seem to increase substantially with the read length when moving from 36 to 75 bp. Indeed, with 75-bp reads and two substitutions, about 20% of the mammalian genome remains unmappable, but the proportion raises to 30% in the case of the fly genome. Even after removing the heterochromatic fraction of the *D.melanogaster* genome –which mainly corresponds to repetitive sequences and could lead to an underestimation of the uniqueness of the genome– the proportion of uniquely mappable positions in fly remains lower if compared to other model genomes.

One hypothesis which could explain, in part, the lower fraction of uniquely mappable regions observed in *D.melanogaster* genome might rely on the different nature and proportion of repetitive elements. Indeed, among repeat elements of class I the fly genome does not contain non-LTR retrotransposons such as SINEs [17], whereas the proportion of LTR retrotransposons constitutes more than one third of the total proportion of repeats (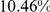 versus 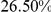; see Table 2). These LTR retrotransposons are found in higher copy number than non-LTR retrotransposons. On the opposite, most of the repetitive sequences in human correspond to non-LTR retrotransposons (Alu sequences) which have a lower number of copies. Thus, the high copy number of LTR retrotransposons in *D.melanogaster* could lead to an over-representation of duplicated 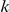-mers that may induce the lower uniqueness observed in this genome.

### Mappability and SNP calling

In this section we show that computing the mappability of a given genomic region can be critical when inferring single nucleotide variants from HTS data. In fact, these are called by looking at the pileup over the same genomic position, i.e. at the set of symbols which are assigned (by the alignment algorithm) to that position.

**Figure 4:**
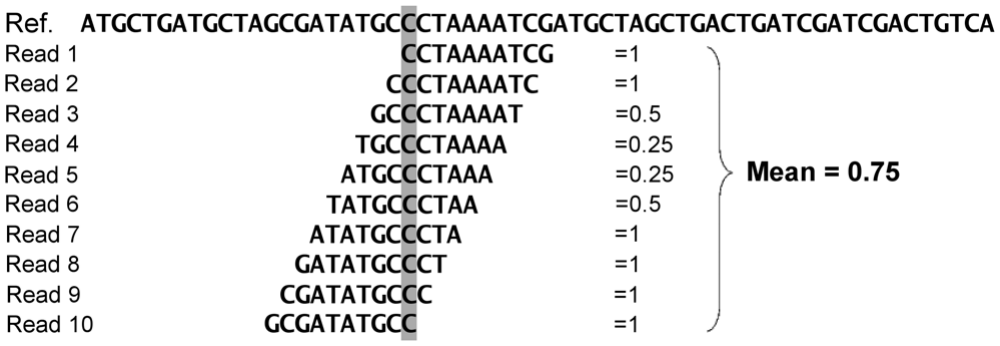
Pileup mappability. The number of all possible 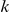-mers covering a particular position of the genome (corresponding to nucleotide C) is equal to 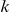 (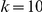 in this example). The average of the mappabilities of the 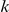-mers can be taken as the *pileup mappability*. Such a quantity represents how mappable would be this position in a pileup of a whole genome sequencing study with reads of length 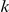.

Before proceeding, we observe that our definition of mappability should be refined if we are to deal with pileups. This is due to the fact that the number of possible 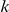-mers covering a particular position of the genome is equal to 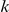, that is to the read length used for sequencing. In other words, when considering a position of the pileup we are not certain about the starting point of the reads which are contributing to it; therefore, using for it the value of mappability as it had been defined so far does not appear to be the best possible strategy. Thus, to estimate how much mappable a pileup position is when using single-end reads, one could take into account the whole set of possible contributing 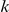-mers, and use the mean of their mappabilities (Figure 4). From now on we will refer to this quantity as to the *pileup mappability*.

In an ideal case, the pileup would either be composed of one kind of nucleotide only, or it would split into two (roughly equal) subsets, if that position corresponds to an heterozygous locus in a diploid organism. In practice things often go differently, for at least two reasons. First of all there are sequencing errors, which introduce spurious nucleotides in the reads; customarily, one dampens the effect of mis-sequencing by scrutinizing the quality score associated to each letter. Futhermore, due to the fact that genomes contains repeats, each alignment suffers from a certain, unescapable, degree of ambiguity.

Namely, when two or more stretches of the sequence under study are very similar to each other, it is not possible to exclude that a read contributing to the pileup in a certain position actually belongs to another portion of the molecule: this leads to occasional mismatches in the alignment, which in turn imply variability in the pileup. It is to quantify the above phenomenon that the concept of (pileup) mappability turns out to be very useful. In fact, if we count the number of symbols different from the reference in the pileup over a certain region of the genome (normalized by the coverage), we expect this quantity to be, on average, inversely related to its uniqueness.

**Figure 5:**
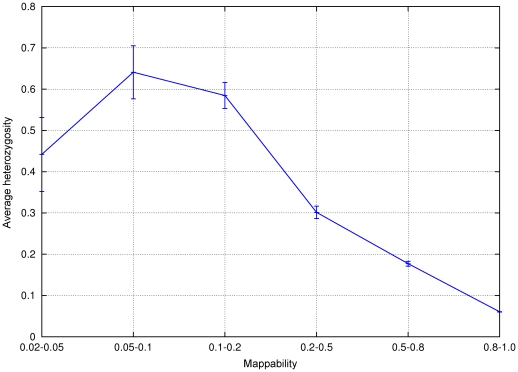
Relation between heterozygosity and pileup mappability. Low-pileup-mappability regions are more prone to show a high value of heterozygosity than those with high mappability. This is due to the spurious contribution of reads which originate from similar regions belonging to the same mappability group. This figure was obtained for *H.sapiens* chromosomes 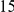 and 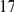 out of an in-house experiment with average coverage 30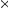.

This is indeed what we observe in Figure 5. To generate it, we considered a pileup computed via the SAMtools pileup utility [18] from reads produced in-house and mapping uniquely to *H.sapiens* chromosomes 15 and 17. We sampled uniformly 100000 positions from each pileup. We then computed the mean heterozygosity (number of symbols in the pileup different from the reference) as a function of the pileup mappability of the position where the read is mapped, grouping together positions with similar levels of mappability.

The figure clearly suggests that to obtain a set of *bona fide* diploid SNPs it could be certainly worth excluding those coming from regions of low pileup mappability.

### Mappability of the projected transcriptome

As we have already pointed out, genome mappability is essential when normalizing counts of reads mapping to the genome in order to obtain quantitative estimates from ChIPSeq experiments. Similarly, transcriptome mappability is also essential when computing normalized counts of transcript abundances after an RNASeq experiment. Here, we sought to apply our method in order to investigate transcriptome mappability.

We use the term transcriptome in the sense being used in RNASeq experiments: a transcript annotation of a reference genome, that is a set of genomic coordinates specifying the exonic structure of transcripts (ideally all known transcripts encoded in the reference genome), or directly the sequence of such transcripts. Most RNASeq protocols map reads to both the genome and the transcriptome, since transcript sequences across splice junctions are not represented in the sequence of the genome.

In this regard, mappability can be understood in two different ways. First, we may compute frequencies by counting 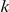-mers in all transcript sequences. Given the high incidence of alternative splicing in eukaryotic transcriptomes [19], mappability obtained in this way is likely to be low. Indeed, exon sequences shared by alternative splice forms will have, by definition, mappability less than one. In fact, deconvolving the originating alternative transcripts of RNASeq reads is one of the most important challenges that need to be overcome to produce accurate quantifications at the alternative transcript level, and a number of methods are being explored towards that end [20], [21].

Alternatively, we can compute frequencies, and from them the mappability, by counting 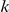-mers in a non-redundant transcriptome in which transcript coordinates are projected onto the genome, and each exon or exon fragment unique to a set of transcripts is considered only once. This is the sense in which we use mappability in our analysis here.

**Figure 6:**
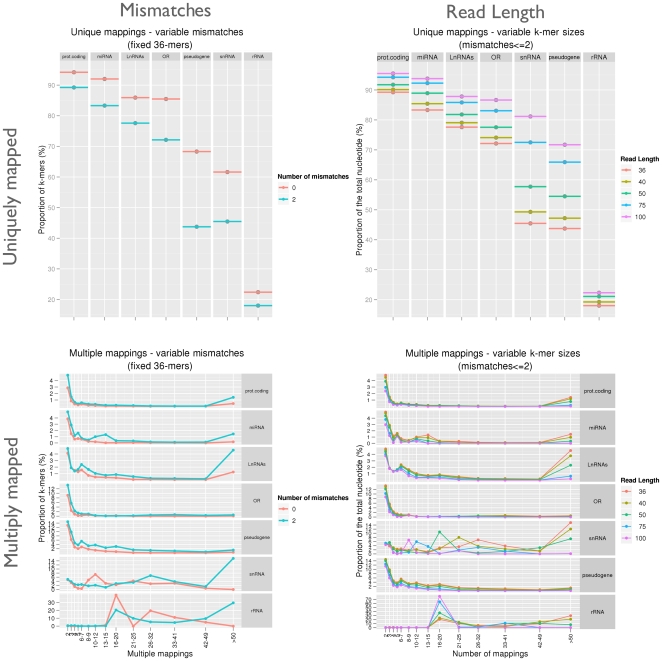
Influence of mismatch values and read lengths on the mappability of the human projected transcriptome as defined by GENCODE [**8**]. For simplicity, we display the proportion of 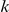-mers having a frequency of 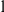 (i.e. uniquely mappable) and those having a frequency 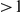 (ambiguous) on the first and second row, respectively. The influence of mismatch number and 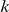-mer lengths are presented in the first and second column, respectively.

As a reference transcriptome dataset, we use the GENCODE annotation of the human genome [8], the most complete transcriptome annotation of this genome currently available. We have partitioned the GENCODE annotated genes into functional sub-classes: protein-coding RNAs (with the Olfactory Receptors, OR, as representative of a superfamily of paralogous genes), long non-coding RNAs (lncRNAs), ribosomal RNAs (rRNAs), pseudogenes, and small non-coding RNAs (considering separately microRNA precursors, miRNAs, and small nuclear RNAs, snRNAs). We have computed the mappability profiles within each of these categories for multiple read lengths and substitution values. Our results appear in Figure 6. For convenience, we separately display the proportion of GENCODE projected exonic 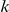-mers having a frequency of one (unique mappings, maximum mappability) from those having a frequency greater than one (multiple mappings, low mappability) for each combination of the tested parameters: 0 or 2 substitutions, and read lengths of 36, 40, 50, 75 and 100 nucleotides.

As expected, the mappability score of a particular 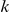-mer in the transcriptome never decreases when increasing the read length; on the other hand, it always tends to decrease when increasing the number of mismatches [22]. However, important differences can be observed between gene classes. For instance, within a mappability profile computed with at least 2 substitutions almost 90% of the protein-coding 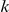-mer exons will be mapped uniquely, even with short sequence reads of 36 bp, whereas this fraction is only 20% for rRNAs, even with longer reads of 100 bp.

It is worth noting that, even if in general protein-coding genes all share a high mappability, large paralogous families are likely to originate smaller fractions of uniquely mappable reads. For instance, the fraction of mappable reads for the roughly 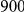 olfactory receptors annotated in GENCODE is at least 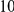
% less than the average of protein-coding genes for all read lengths and number of substitutions considered. This and similar cases will originate a clear bias whenever transcript expression measurements of paralog genes are attempted during RNASeq experiments.

Pseudo-genes, which still share a relevant sequence similarity with their parent genes, show an even lower mappable fraction. Interestingly, the highest variation observed between the mappability computed with 0 and 2 substitutions concerns pseudogenes (from 69% to 44%, respectively). This observation might be due to the fact that duplicated pseudogenes present in many copies escape purifying selection, and thus tend to accumulate more mutations if compared to their parent genes.

**Figure 7:**
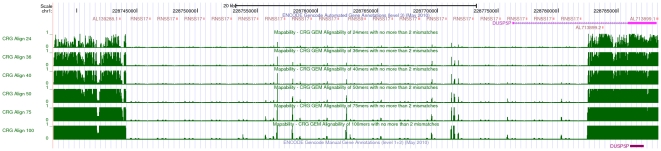
Cluster of 5S rRNAs on human chr1 exhibiting a very low mappability profile. This locus explains the peaks observed for annotated rRNAs in the frequency range 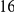-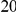.

The long non-coding RNAs (lncRNAs) [23], [24] also seem to be less unique than protein-coding sequences; interestingly, they contain a significant proportion of nucleotide mapping more than 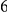-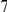 times in the genome, probably reflecting their tendency to be enriched in repetitive elements such as SINEs or LINEs [25]. Finally, the short non-coding RNAs (separated into miRNAs and snRNAs) present distinct mappability profiles which are directly related to the presence of subfamilies and/or derived pseudo-copies within each class. For instance, our manual investigation of the peculiar peak in the proportion of rRNA 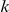-mers that could be mapped 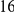-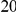 times (see Figure 7) showed that the phenomenon is due to a the sub-family of 5S rRNAs belonging to the large subunit of the ribosome, clustered together on chromosome 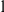.

### Refining expression level measure derived from RNASeq data

Transcriptome mappability can be used to produce more accurate estimates of transcript abundances from RNASeq experiments. As we have seen, some gene classes and families are characterized by low sequence uniqueness, meaning that reads mapping to their sequence are likely to map to (many) other locations in the genome/transcriptome. Different RNASeq mapping and quantification strategies deal with this issue in different ways. They may simply ignore reads that either map to multiple locations [26] or fall in low-mappability regions [27]. They may select one mapping location (or a few), either randomly [28] or using additional context-based information – such as the mapping of mate pairs in paired end sequencing, or the density of mappings in the neighborhood [29], [30]. Finally, some quantification strategies keep all mapping locations (within the capacities of the used mapping algorithm) and possibly, during the quantification step, employ a statistical model (such as Poisson distribution, Bayesian networks, maximum likelihood, etc.) to infer read counts among all transcripts [1], [20], [31]–[33].

A widespread measure of exon, transcript and gene abundance in RNASeq experiments is the so-called RPKM (Reads Per Kilobase of exon per Million mapped reads) [30]. If reads mapping to multiple locations are simply discarded, as it is often the case, RPKM may underestimate the expression of genes belonging to conserved paralogous families.

For instance, let us assume that two genes exist, both having exactly the same length and originating exactly the same number of reads, but the first being uniquely mappable on its entire length (mappability equal to one everywhere), while the second is uniquely mappabile only in half of its length. Insofar as non-unique reads are ignored, if the two genes are equally expressed the RPKM of the second one will artificially turn out to be the half of that of the first one.

**Figure 8:**
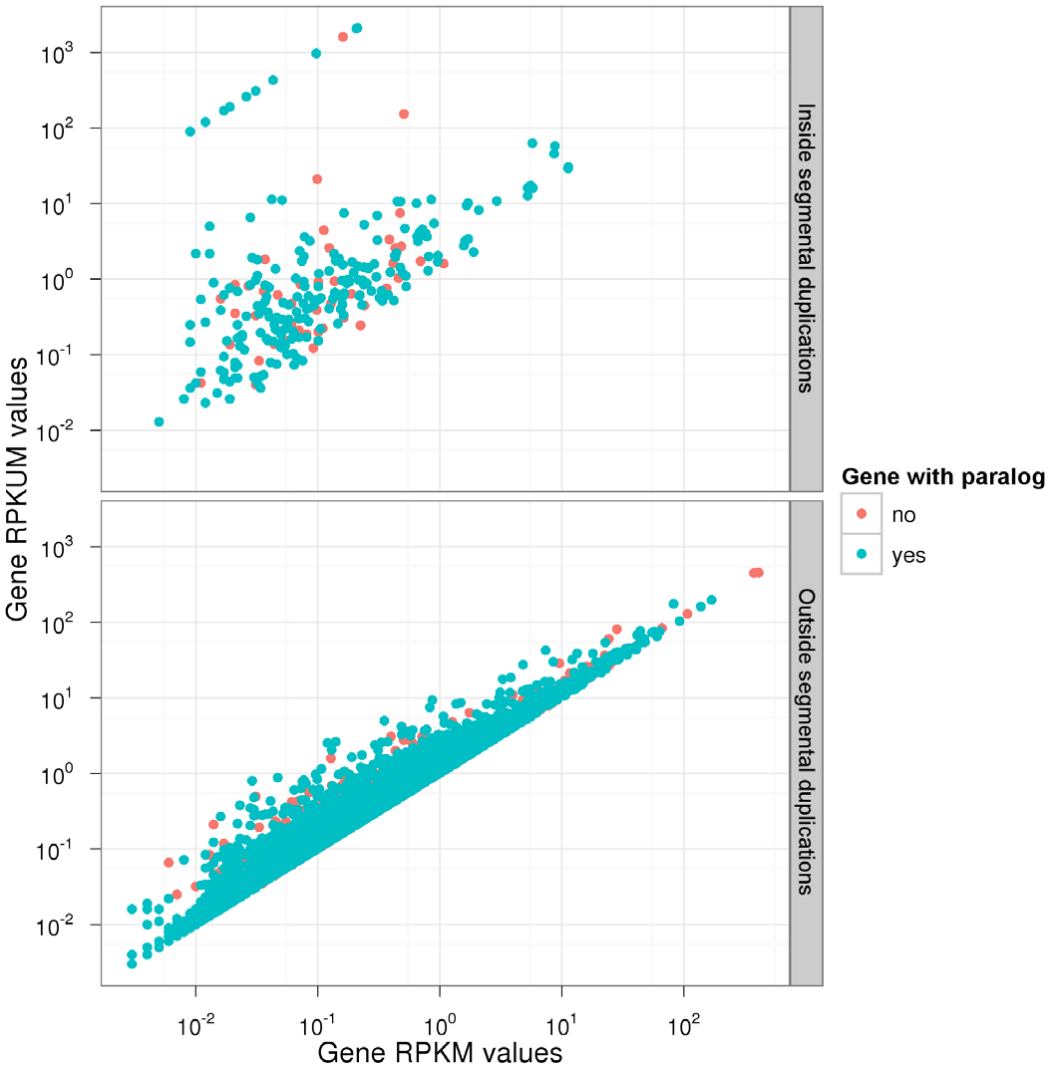
Comparison of Gencode protein-coding genes RPKM and RPKUM expression values as measured in brain tissue (data from [**19**]). Both axis are log-scaled, and each dot represents a protein-coding gene with or without annotated paralogous genes (in green and red, respectively). Protein coding genes totally or partially included in segmental duplications are presented in the top panel, whereas those not overlapping segmental duplications are shown in the bottom panel. The figure illustrates the importance of taking into account the mappability information in order not to underestimate expression level. Without mappability correction, two main reasons are shown to introduce a bias in the quantification of expression levels: gene having paralogs, and genes overlapping segmental duplications.

In fact, if reads mapping to multiple locations are discarded, only locations with mappability of one can actually contribute to the normalization of expression. In such a case, therefore, we suggest to compute RPKM considering only the fraction of uniquely mappable positions of the feature being quantified (exon, transcript, gene). More specifically, we suggest to compute instead the Reads Per Kilobase of Unique exon per Million mapped reads (RPKUM). Figure 8 illustrates the comparison between RPKM and RPKUM values for GENCODE protein-coding genes. Both expression measures were computed using an RNASeq experiment of the human brain transcriptome which produced 32-bp reads [19]. In this analysis, we have mapped the reads to both genome and transcriptome using GEM [2], and allowing for two mismatches.

Separately, we produced the mappability profile of the human genome with identical mapping parameters (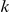-mer length 32, and at most 2 mismatches). Then, for each protein-coding gene annotated by GENCODE, we computed the RPKM and the RPKUM as defined previously. As one can readily observe, for a substantial number of genes the RPKUM measure is significantly higher than the corresponding RPKM, and for few of them the differences may be of up to several orders of magnitude (Figure 8). Clearly, genes exhibiting the highest difference between RPKM and RPKUM typically belong to either of the following two families:

1. genes partially or totally included a segmental duplication as defined in [34] (top panel of Figure 8)
2. genes having at least one paralog (green dots) as identified by the Ensembl Compara database [35].

**Figure 9:**
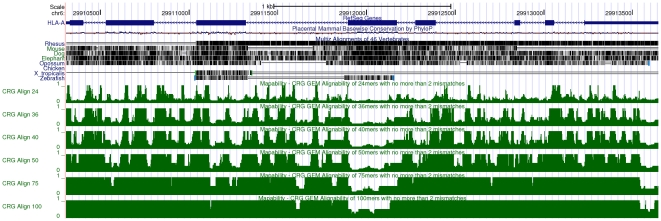
Influence of paralogous genes on the mappability scores: the example of the HLA-A gene. The HLA-A gene is part of the Major Histocompatibility Complex (MHC) involving a large gene family with numerous paralogs. This screenshot of the UCSC genome browser (with the six mappability tracks in green) illustrates the low uniqueness of the HLA-A gene (especially, its exon 4) which could render its targeting by RNASeq difficult (if only uniquely mapping reads are considered).

A striking example is the HLA-A gene (Human Leucocyte Antigen, class I, A) involved in the major histocompatibility complex, which has many paralogs (Figure 9). More generally, amongst the top 1000 genes exhibiting the highest variations between RPKM and RPKUM, 741 (74,1%) have at least one paralog gene.

It should be noted that the importance of computing the uniquely mappable area of a transcript in order to refine its RNA abundance quantification is gaining more and more attention: for instance, a very sophisticated strategy to accurately perform this task has been recently presented in [36]. However, such a strategy does not rely on the explicit pre-computation of the mappability, nor it takes mismatches into account when computing the length of mappable regions: using our algorithm as the first step of that method might lead to even better results.

If the chosen RNASeq mapping strategy consists of selecting one mapping location among the many possible ones, mappability can still be used as an additional criterion to help in the selection. Finally, if the contribution of mapped reads to the quantification of transcriptional features (exons, transcripts, genes, etc.) is weighted by the number of mapping locations, the frequencies as computed by our method may also be relevant. Indeed, being not exhaustive, most mapping algorithms are unable to report the exact number of existing matches, and hence the exact frequency value. Thus the frequencies produced by our method (provided that a suitable value for 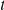 is chosen, as explained in Section Methods) would produce more accurate corrections.

#### Mapping and mappability: a complicated relation

**Figure 10:**
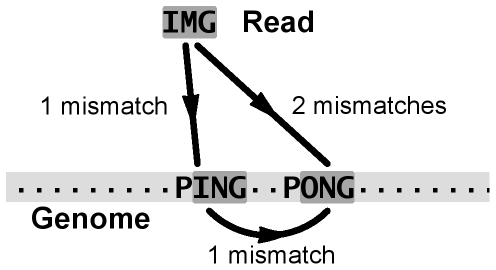
Read mapping and mappability are different concepts: there is no straightforward relation between the number of times a read matches the genome and the mappability of the regions it maps to. Within an edit distance of 1 mismatch, the sequence IMG maps uniquely to location ING in the schematic genome “......PING-PONG.....”. However, the matched position is not unique in the genome, since considering 1 mismatch it has a frequency of 2 due to location ONG.

One fact in need of being emphasized is that, when mapping with mismatches, the relation between mapping uniqueness and mappability is rather complex. Given some edit distance greater than 0, indeed, some counterintuitive situations might arise, where a read which does not occur in the genome maps uniquely (within the specified edit distance) to a repetitive location having a low mappability. This fact is illustrated with the toy example of Figure 10, where one substitution is used as the maximum allowed edit distance both for computing the mappability and for mapping. With such a choice of parameters the read will map to only one location; yet this position is not unique in the genome, since it has a frequency of 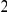 (or, equivalently, a mappability score of 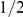). A similar phenomenon happens each time the placement of sequencing errors present in the read forbids the mapping to all copies of a repeated region but one.

In conclusion, knowing that a read maps uniquely to a location is in general *not* enough to establish, when mismatches are considered, that such a location is unique. In this case, a better indicator for the “uniqueness of the read” is likely to be the theoretical mappability of the region, which has to have been computed separately. The existence of this problem is often overlooked.

Strictly speaking, this not-so-straightforward connection also complicates the (re)definition of expression measures able to take correctly into account the reduced number of unique reads in repetitive loci; however, neither this observation diminishes the need for such measures, nor it makes less natural and appealing the definition of the RPKUM measure previously presented earlier.

### Mappability of paired-end reads

In this last section, we examine how the mappability information can be used together with paired-end/mate-pair sequencing to improve the design of an HTS experiment. In particular, we show that when the mappability is known it is possible to tune the insert size in order to maximize the number of sequencing pairs which one will be able to rescue by resorting to the uniqueness of either end.

**Figure 11:**
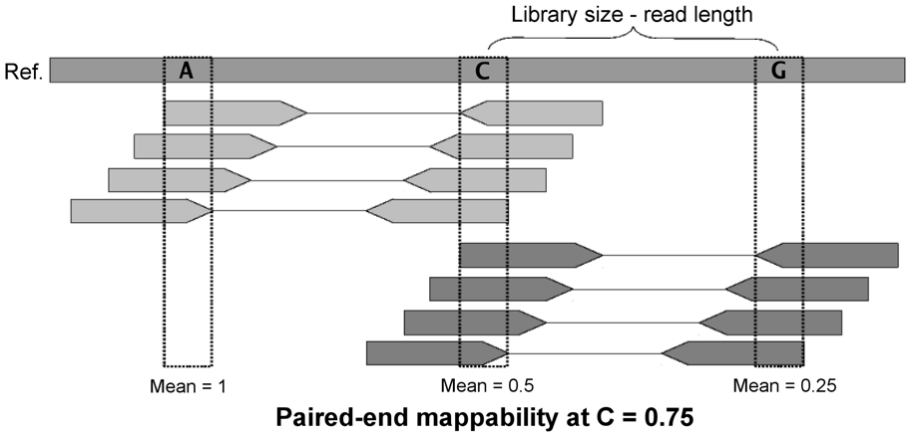
Schematic representation of the computation of the paired-end mappability. In this example the average of the single-end mappabilities at the target position (base C) is bigger than the average of the single-end mappabilities at one of the pairs (base A). Hence the resulting paired-end mappability will be the average of the mean mappabilities at C and A.

When using paired-end reads (or mate-pairs), the mapping information of one end can be used to discard spurious mapping positions of the other end if one takes into account the expected distance between ends imposed by the library size used for sequencing. In consequence, when sequencing with a paired-end type strategy, the *paired-end mappability* of a position 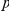 will be function of both its own single-end mappability and the mappabilities of the positions located at 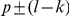, being 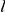 the library size (see Figure 11).

To facilitate the analysis, we have assumed that the standard deviation of the fragment size in each library is zero (that is, all the fragments in the library are having exactly the same size, and hence all the pairs the same distance).

Given the assumptions just presented, it is straightforward to conclude that exactly three cases are possible, as follows (in Figure 11 we illustrate them for the case of paired-end pileup mappability):

1. the single-end mappability of the target position is bigger than, or equal to, the mappabilities of the two possible pairs: the paired-end mappability is not affected by the mappability of the pairs
2. the single-end mappability of the target position is smaller than the mappabilities of one of the pairs: as the new mappability of the target, one can take the average of the single-end mappability of the target and the single-end mappability of that pair
3. the single-end mappability of the target positon is smaller than the mappabilities of the two possible pairs: as the new mappability of the target, one can take the average of the single-end mappabilities of the two possible pairs.

The latter two cases might allow –depending on the single-end mappability of the various loci– to rescue reads which are not by themselves uniquely mappable.

It should be noted that in our calculations we did not take into account paired-end configurations which, while not being unique at any of the pairs, could be still rescued due to the fact that only one of the possible matches for the pair is having the expected insert size. On the contrary, we might be overestimating the mappability of the flanks of regions having long series of tandem repeats: in such a case, a big standard deviation in the sizes of the fragments belonging to a library would complicate the process of identifying a single compatible pair by the expected size of the insert region between the ends, as the problematic reads will have alternative mapping positions very close to each other.

**Figure 12:**
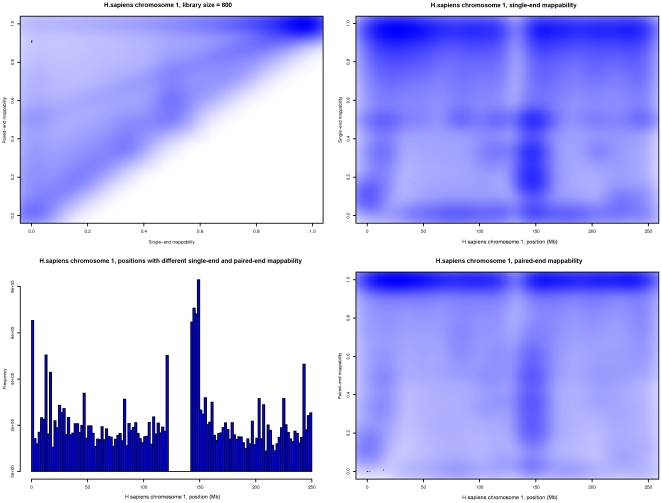
Behavior of pileup single-end and paired-end mappabilities at different loci of human chromosome 1 (HSA1). Parameters used to generate this example were: 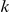-mer length 100, 2 mismatches and a library size of 800 bases. Top left: Heatmap of the number of locations in HSA1 as a function of their single-end and paired-end mappabilities. Bottom left: Histogram of the number of locations in HSA1 that show different single-end and paired-end mappabilities, plotted versus their position along the chromosome. Top right: Heatmap of the number of locations in HSA1 as a function of their single-end mappability and their position along the chromosome. Bottom right: Heatmap of the number of locations in HSA1 as a function of their paired-end mappability and their position along the chromosome.

In Figure 12 we present the results of a comparison of single-end and paired-end mappabilities for human chromosome 1 (HSA1) when using a library size of 800 bp. On the heatmap plots one can spot that even when using 100-bp reads the increase in unique mappability can be considerable if the pair information is integrated. Another interesting feature is the distribution of the positions of HSA1 having different single- and paired-end mappabilities: we can clearly identify the centromere position as the one where both mappabilities are the same (and close to zero).

**Figure 13:**
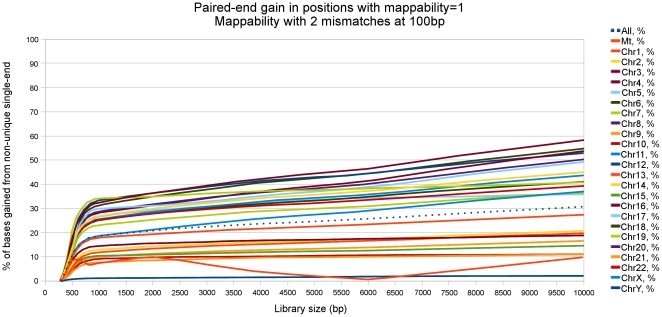
Proportion of completely rescuable positions for all human chromosomes. In this figure we only consider positions having a single-end mappability greater than 1, and for different library sizes (300, 400, 500, 600, 700, 800, 900, 1000, 1500, 2000, 4000, 6000, 8000 and 10000 bp) we plot the fraction of locations which could be rescued by taking advantage of the fact that they have a paired-end mappability equal to one.

Additionally, in order to evaluate the importance of using paired-end information when processing the results of read mapping, we have estimated the single- and paired-end mappability of 100-bp reads for a set of library sizes (300, 400, 500, 600, 700, 800, 900, 1000, 1500, 2000, 4000, 6000, 8000 and 10000 bp) along the whole human genome. To this end, we have estimated which proportion of positions having a non-1 single-end mappability can be rescued completely owing to the fact that both possible pairs in a paired-end experiment are unique. Figure 13 clearly shows that when increasing the library size also the proportion of reads rescued with this method increases; for large library sizes and some chromosomes (e.g. 3, 4 or 6), such proportion can be higher than 50%.

Remarkably, at small library sizes (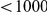 bp) the fraction of rescued reads increases very fast with the distance between the ends. At this scale, the improvement in uniqueness is expected to happen in short regions of the genome (like transposons) which can be seen as unique if they are smaller than the library size, and such that the sequence context around them is itself unique. On the contrary, while for bigger library sizes the percentage of rescued reads keeps growing, the slope of the improvement is much smaller. This result would seem to indicate that in the latter case the repetitive regions we are trying to rescue are much bigger (for instance, this could be the predominant situation for chromosome Y, where the advantage given by such a rescuing strategy turns out to be minimal).

## Discussion

In this work, we explore the mappability concept with unprecedented detail, presenting a fast algorithm to compute a well-behaved approximation of the mappability at the level of an entire mammalian genome, even when mismatches are allowed or when small read lengths are used. Our program is freely available, and can be easily used to construct mappability profiles of any given genome. Our visualization tracks of human and mouse mappability profiles are already accessible through the official UCSC genome browser, and more could be uploaded as custom tracks for different model organisms. Auxiliary tracks can be easily derived from the existing ones to account, for instance, for CG-content sequencing bias.

The analysis of the uniqueness of a genome (i.e. the proportion of 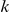-mers having a mappability score of 1) for four model organisms (human, mouse, fly and nematode) computed with up to 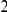 substitutions revealed a more complex architecture than anticipated. Regions of the genome that are not uniquely mappable correlate not only with the global proportion of repetitive sequences but also, more importantly, with the nature (and hence, the number of copies) of these repeats.

Computing the mappability of a genome is very useful in ChIPSeq experiments, in order to provide a suitable normalization when peaks are scored. In a given RNASeq experiment, calculating *a priori* the mappability sheds light on regions which will not be easily accessible if multiple mappings are discarded; it could also help to design a better experiment, in particular whenever the main goal is either to exploit most of the biological signal, or to access a specific feature of a genome.

Indeed, we also showed that mappability profiles vary significantly depending on the type of functional element studied and the parameters used (read length and/or number of mismatches). In particular, the analysis of the mappability profiles of gene families (like the olfactory receptors) and pseudo-genes shows that even long HTS reads are not enough to make some features easily accessible: just using a longer read length may not be enough by itself to completely eliminate the ambiguity which arises from the repetitive nature of some interesting features of the genome.

The connection with the design and the analysis of HTS experiments at the level of the single locus is therefore straightforward. We further emphasized it by examining how mappability impacts the study of single-nucleotide polimorphisms, and how it relates to paired-end sequencing schemes.

Finally, one could note that the systematic fast computation of mappability may be used in various situations of common interest in biology other than those related to the analysis of HTS data – typical examples being the identification of interesting repeated motifs, or the refinement of primer design. Overall, we believe the present work still far from being exhaustive: more and more practical applications of the study of sequence mappability will certainly follow in the future.
